# Programmable SCOW Mesh Silicon Photonic Processor for Linear Unitary Operator

**DOI:** 10.3390/mi10100646

**Published:** 2019-09-26

**Authors:** Liangjun Lu, Linjie Zhou, Jianping Chen

**Affiliations:** State Key Laboratory of Advanced Optical Communication Systems and Networks, Shanghai Institute for Advanced Communication and Data Science, Shanghai Key Lab of Navigation and Location Services, Department of Electronic Engineering, Shanghai Jiao Tong University, Shanghai 200240, China; ljzhou@sjtu.edu.cn (L.Z.); jpchen62@sjtu.edu.cn (J.C.)

**Keywords:** photonic processors, unitary transformation, silicon photonics

## Abstract

Universal unitary multiport interferometers (UMIs) can perform any arbitrary unitary transformation to a vector of input optical modes, which are essential for a wide range of applications. Most UMIs are realized by fixed photonic circuits with a triangular or a rectangular architecture. Here, we present the implementation of an *N* × *N* rectangular UMI with a programmable photonic processor based on two-dimensional meshes of self-coupled optical waveguide (SCOW) resonant structures. Our architecture shows a high tolerance to the unbalanced loss upon interference. This work enriches the functionality of the SCOW mesh photonic processors, which are promising for field-programmable photonic arrays.

## 1. Introduction

Photonic integration has attracted increasing interest in the potential of extensive reduction of size, weight, and power (SWaP). Silicon photonics is a promising solution for complex integrated photonic systems with low cost and high integration density, due to the advantages of complementary metal-oxide-semiconductor (CMOS) compatible processing and high refractive index contrast. Lots of silicon-based photonic integrated circuits (PICs) have been demonstrated in a wide variety of applications. Among them, unitary multiport interferometers (UMIs) are now receiving more and more attention. UMIs can implement various arbitrary unitary transformation, addressing a wide range of applications including mode-division (de)multiplexing [1,2], photonic deep machine learning [3], quantum particle simulation [4], and quantum signal processing [5,6]. Besides, a UMI can be extended to any *M* × *N* linear (non-unitary) transformation. The most common architecture for UMIs is based on a specific triangular mesh of 2 × 2 couplers and phase shifters, proposed by Reck et al. [7]. Recently, a silicon 4 × 4 photonic UMI has been experimentally demonstrated [8]. An optimal design with a more symmetrical rectangular mesh for the UMIs shows the superior performance of less optical depth and more robustness to optical losses compared with Reck’s architecture [9].

Inspired by the concept of Field Programmable Gate Arrays (FPGAs), general photonic processors, which can be programmed to perform multiple photonic processing functions by using the same hardware configuration, have the merits of higher flexibility and more cost-effectiveness compared with application-specific PICs (ASPICs) [10,11,12]. Several elegant programmable photonic processors have been proposed and demonstrated with square [13], hexagonal [14], or triangular [15] mesh networks. These processors show versatile functionalities in optical and microwave photonics signal processing [16,17,18]. The method and algorithm for setting up mesh networks composed of non-ideal components have also been proposed [19,20,21]. Recently, Perez et al. realized a rectangular UMI with the hexagonal mesh processor by properly setting tunable waveguide couplers. This work, in turn, verifies the reconfiguration capability of the hexagonal meshes [22].

In our previous work, we proposed a reconfigurable photonic processor based on a mesh of self-coupled optical waveguide (SCOW) resonators [23]. This architecture has the advantages of high scalability and versatile configurations, which can be programmed to various optical components like ring resonators, Mach–Zehnder interferometers (MZIs), Fabry–Perot (FP) cavities, and also more complicated structures composed of these basic components. These structures are key components in various photonic processing. In this work, we extend the functionality of the SCOW-mesh photonic processors to an arbitrary unitary operator. We present, in detail, the implementation method as well as the synthesis algorithm for an *N* × *N* rectangular UMI. These results show the capability of the programmable SCOW mesh processors.

## 2. Materials and Methods 

**Figure 1:**
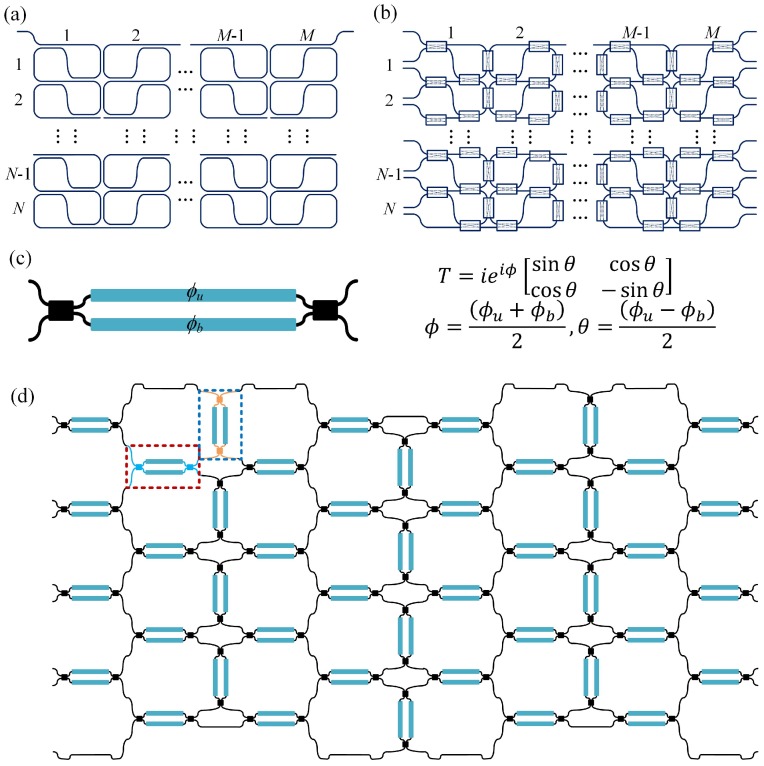
(**a**) Schematic of an array of *N* × *M* SCOW resonators placed in a two-dimensional mesh. (**b**) Schematic of an *N* × *M* SCOW mesh photonic processor with tunable couplers. (**c**) Structure of a tunable coupler based on a 2 × 2 MZI. *ϕ_u_* and *ϕ_b_* are phases of the upper and bottom arms of the MZI, respectively. *T* is the transfer matrix of the MZI. (**d**) Schematic implementation of a 4 × 4 SCOW mesh processor constructed by 2 × 2 MZI tunable couplers. The green dashed circle represents a SCOW unit composed of six MZIs placed in the vertical and horizontal directions. All the vertical MZIs (blue dashed rectangle) and the horizontal MZIs (red dashed rectangle) are designed with the same equivalent optical path length.

The SCOW resonator is formed by bending a single waveguide twice to form two directional couplers (DCs) at the input and output ports [24,25]. A single-stage SCOW resonator has versatile spectral responses from both transmission and reflection sides, depending on the coupling coefficients of these two DCs. We can also realize a two-dimensional array of SCOW resonators as illustrated in Figure 1a. In the vertical direction, two contiguous SCOWs share two DCs, while in the lateral direction, the two adjacent SCOWs are placed closely to form an additional DC.

To realize a reconfigurable photonic processor, we replace the passive couplers with tunable couplers (TCs), as shown in Figure 1b [26,27]. By adjusting the coupling states of the TCs, the SCOW mesh processor can be reconfigured to various optical structures with versatile spectral responses, which can accomplish multiple signal processing tasks. For an *N* × *M* photonic processor, there are totally 3*MN* − *N* TCs. As shown in Figure 1b, we break the connecting waveguides at the right and left edges of the processor to form multiple input and output ports. The TC can be formed by a 2 × 2 MZI, as depicted in Figure 1c. According to the transfer matrix, the differential phase between the two arms of the MZI, *θ* = (*ϕ_u_* − *ϕ_b_*)/2, decides the coupling coefficient of the TC, while the common phase, *ϕ* = (*ϕ_u_* + *ϕ_b_*)/2, decides the phases of the output ports. When the two MZI arms are in phase (*θ* = 0), light from the input port is fully transmitted to the cross port, which is referred to as cross-state. In the case where the phase difference of two arms is equal to π (*θ* = π/2), the MZI is changed to the bar-state. For these two specific cases, the MZI works as parallel or crossed waveguides with their phases controlled by *ϕ*, which can be recognized as phase shifters. 

For universal unitary transformation, any two interference optical paths must have an equal path length. Therefore, the MZI elements should be designed with the same equivalent optical path length. Figure 1d shows an example of a 4 × 4 SCOW mesh processor for UMI application. The green dashed circle represents a SCOW unit consisting of six directly-connected MZIs without any extra connecting waveguide. The MZIs are arranged in either vertical or horizontal directions. In order to get an equal optical path length, these two kinds of MZIs are slightly different at the input and output ports as shown the red and blue dashed rectangles in Figure 1d. At the top and bottom edges, one of the MZIs in the SCOW unit is replaced by a propagation waveguide with an equal optical length. In this case, we can easily construct a rectangular UMI for arbitrary unitary transformation applications even when used with a pulsed signal.

## 3. Results

**Figure 2:**
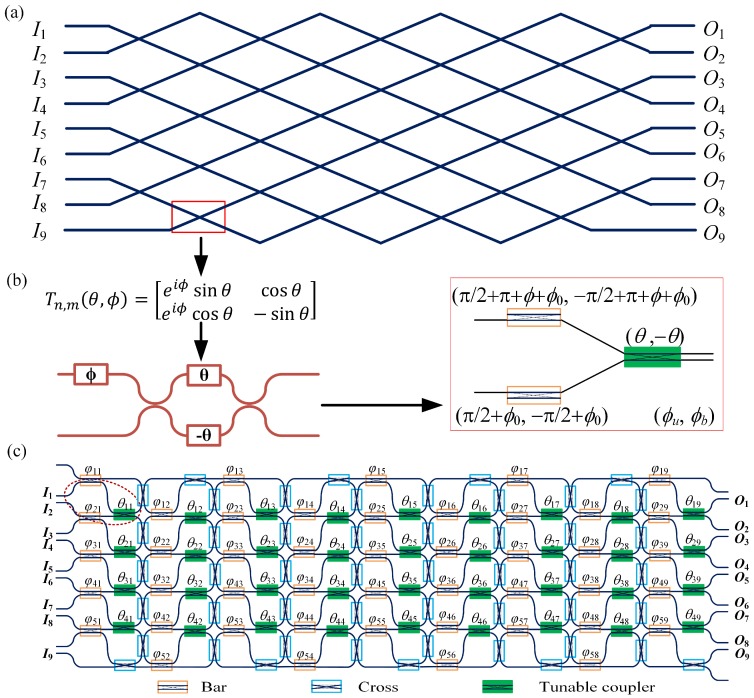
(**a**) A 9 × 9 rectangular UMI as proposed in [9]. (**b**) The crossing in the rectangular UMI is represented by a transfer matrix, *T_m,n_*(*θ*, *ϕ*), implemented by a 2 × 2 coupler and a phase shifter. It can be equivalently implemented by three MZIs in the SCOW-based mesh network. (**c**) Equivalent implementation of the 9 × 9 rectangular UMI using the SCOW-based photonic processor.

Here, we present the implementation of the SCOW mesh photonic processor as a rectangular UMI. Figure 2a shows an example of the 9 × 9 rectangular UMI proposed by Clements et al. [9]. There are 36 crossings in the UMI. Each crossing represents a two-path interferometer. It corresponds to a variable beam splitter described by an optical field transfer matrix, *T_m,n_*(*θ*, *ϕ*), as depicted in Figure 2b. It can be implemented by an MZI with an additional phase shifter at one input port. In our proposed processor, the variable beam splitter can be constructed by three MZIs as illustrated in Figure 2b. The left two MZIs are considered as input ports of the variable beam splitter, which are at the bar-state. These two MZIs also serve as two phase shifters with target phases of *ϕ* + *ϕ*_0_ and *ϕ*_0_. To be mentioned, for an MZI at the bar-state, the bottom path has an additional π phase shift compared with the upper path, as shown in Figure 1c. Therefore, the applied phase (*ϕ_u_*, *ϕ_b_*) of the upper MZI has an extra common phase shift of π for the target phase of *ϕ* + *ϕ*_0_. That is very important in the implementation of the rectangular UMI, where we share the bar-state MZI as the phase shifters for two adjacent variable beam splitters in the same column, as indicated by the dashed circles in Figure 2c. The right MZI works as the TC. Therefore, at the output ports of the right MZI, the transfer matrix is:

(1) $$M=ie^{i\phi_{0}}\left[\begin{array}{cc} e^{i\phi }\sin\theta & \cos\theta \\ e^{i\phi }\cos\theta & -\sin\theta \end{array}\right]$$

From Equation (1), we can see that *M* equals to *i*exp(*iϕ*_0_)*T_m,n_*(*θ*, *ϕ*). The common phase item *i*exp(*iϕ*_0_) only brings a phase to the final diagonal matrix, which can be omitted.

Figure 2c shows the implementation of a 9 × 9 rectangular UMI using the proposed photonic processor. The processor is based on a 5 × 9 SCOW mesh, which incorporates 130 MZIs in total. We use simple rectangles to represent all MZIs and different colors for MZI working states. The MZI design parameters are the same as in Figure 1d. The optical path length of one MZI is defined as *L_mzi_*. There are four variable beam splitters in every column, which are all constructed by three MZIs. Each two adjacent beam splitters share a bar-sate MZI. Therefore, in every column, there are five bar-state MZIs and 4 MZIs working as the TCs. The rest of the MZIs are implemented as cross-connecting waveguides once they are tuned to the cross-state. The target phases *ϕ* and *θ* of the variable beam splitter in the *d^th^* row and the *q^th^* column of the two-dimensional SCOW mesh are denoted as *ϕ_dq_* and *θ_dq_* (*d* = 1, 2, 3, 4; *q* = 1, 2,…, 9). These target phases can be calculated by the decomposition method proposed by Clements et al. [9], which will be presented later. The phases of the bar-state MZIs are denoted as *φ_pq_* (*p* = 1, 2,…, 5), as labeled in Figure 2c. As the phases of all the 5 bar-state MZIs in one column can be tuned independently, it is sufficient to determine the 4 independent phases in the variable beam splitters as required for practical application.

For universal unitary applications, any interferometer in the UMI must have an equal path length. We can see that in the first column, each interferometer has an equal path length of 2*L_mzi_*. In the second column, the first three interferometers have two interferential optical paths with one shared cross-state MZI and one independent bar-state MZI. The optical path length is 3*L_mzi_*. For the fourth interferometer, light from *I*_9_ passes two cross-state MZIs, a bar-state MZI and a propagation waveguide with a length of *L_mzi_* before interfering with another path. The other optical path includes the first stage interferometer, a cross-state MZI, and a bar-state MZI, which has the same path length as that for interference. For the first interferometer of the third column, the upper optical path originates from the first column interferometer and then passes one propagation waveguide and three MZIs with 4*L_mzi_* equivalent path, which is the same as the bottom optical path. It is easy to verify that the rest of the interferometers also have equal path lengths, which proves the functionality of our SCOW-mesh photonic processor. 

**Figure 3:**
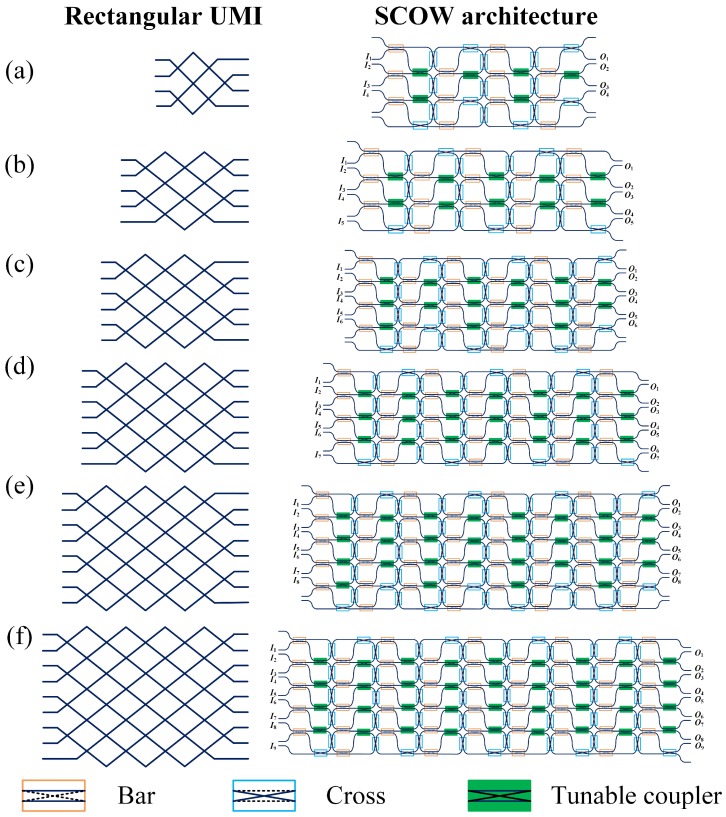
Schematics of the rectangular UMIs and the equivalent implementations using SCOW-based photonic processors. The scales of the UMIs are (**a**) 4 × 4, (**b**) 5 × 5, (**c**) 6 × 6, (**d**) 7 × 7, (**e**) 8 × 8, and (**f**) 9 × 9.

Our SCOW-based processors can be easily programmed to an arbitrary rectangular UMI with such an implementation method. For an *N* × *N* rectangular UMI, the minimum number of variable beam splitters is *N*(*N* − 1)/2. Figure 3 shows the schematics of various rectangular UMIs and the corresponding implementations with SCOW-based processors. The number of columns in the SCOW processor linearly increases with *N*, while the number of rows equals to *mod* ((*N* + 2)/2). In each SCOW, the left MZI is configured to the bar-state. When *N* is odd, *N* − 1 of the right MZIs in each column work as TCs, while the other MZI is at the cross-state. When *N* is even, the number of cross-state MZIs increases to two for each even column. The rest of the MZIs are implemented to the cross-state. The required numbers of MZIs are listed in Table 1.

The synthesis procedure of the rectangular UMI with the hexagonal mesh processor has been proposed by Perez et al. [22]. The implementation procedure of an *N* × *N* UMI for the SCOW-based processor is similar to that of hexagonal meshes. The algorithm proceeds by nulling successive matrix elements starting from the targeted unitary *N* × *N* matrix *U*. Depending on the location of the element *U*(*n*, *m*) to be canceled, a row or column combination of the matrix is required [9,22]. If *N* – *n* − *m* is odd, any target element in column *n* or *m* of *U* can be nulled by multiplying *U* from the right by a *T_m_*_,*n*−1_ matrix. While if *N* − *n* − *m* is even, then, any element in rows *n* or *m* can be nulled by *T_m,n_U*. For the case of the proposed processor, *T_m,n_* represents:

(2) $$T_{m,n}=\left[\begin{array}{cccccccc} 1 & 0 & \ldots & \ldots & \ldots & \ldots & \ldots & 0\\ 0 & 1 & & & & & & .\\ . & & . & & & & & .\\ . & & & e^{i\phi }\sin\theta & \cos\theta & & & .\\ . & & & e^{i\phi }\cos\theta & -\sin\theta & & & .\\ . & & & & & . & & .\\ . & & & & & & 1 & 0\\ 0 & \ldots & \ldots & \ldots & \ldots & \ldots & 0 & 1 \end{array}\right]$$

According to [22], the values of *θ* and *ϕ* of the corresponding matrix *T* are given as:

(3) $$\begin{array}{c} \theta =a\sin\left|\sqrt{\frac{{\left|U\left(n,m\right)\right|}^{2}}{{\left|U\left(n,m\right)\right|}^{2}+{\left|U\left(n,m+1\right)\right|}^{2}}}\right|\\ \phi =∠U\left(n,m\right)-∠U\left(n,m+1\right)-\pi \end{array}\;(when\;N-n-m\;is\;\mathrm{odd})$$

(4) $$\begin{array}{c} \theta =a\sin\left|\sqrt{\frac{{\left|U\left(n,m\right)\right|}^{2}}{{\left|U\left(n,m\right)\right|}^{2}+{\left|U\left(n-1,m\right)\right|}^{2}}}\right|\\ \phi =∠U\left(n,m\right)-∠U\left(n-1,m\right) \end{array}\;(when\;N-n-m\;is\;\mathrm{even})$$

With the calculated *θ* and *ϕ* of each variable beam splitter, we can deduce the applied phases to the MZI arms. The target phases *ϕ* and *θ* of the beam splitter in the *d^th^* row and the *q^th^* column of the two-dimensional SCOW mesh are defined as *ϕ_dq_* and *θ_dq_* (*d* = 1, 2, 3, mod ((*N* − 1)/2), *q* = 1, 2,…, *N*). The applied phases of the MZI TCs are *θ_dq_* and −*θ_dq_* for the two MZI arms, respectively. Each bar-state MZI is shared by two beam splitters, which is different from that of hexagonal meshes. The bar-state MZIs are defined as *φ_pq_* (*p* = 1, 2,…, mod ((*N* − 1)/2) + 1). It means that the applied phases on the two arms of the bar-state MZIs are π/2 + *φ_pq_* and −π/2 + *φ_pq_*, respectively. As there are enough bar-state MZIs in each column independently determining the target phases of the variable beam splitters, we can easily decide the phases of all the bar-state MZIs one by one from the former bar-state MZI with the relation of *φ_p+1,q_* = *φ_pq_* + π − *ϕ_dq_*. Once we fix the first bar-sate MZI in each column, all the other bar-state MZIs can be decided. Therefore, with the implementation method, the SCOW mesh photonic processors can be easily programmed to an arbitrary rectangular UMI, which shows the reconfiguration capability of the SCOW architecture. 

**Figure 4:**
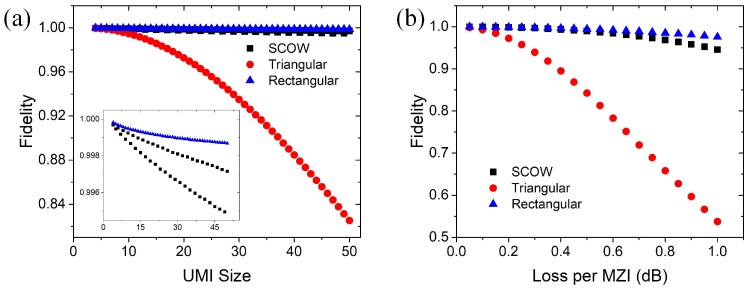
(**a**) Simulated average fidelity as a function of UMI size when the MZIs have a constant loss of 0.2 dB. The inset shows the close-up view of the fidelity of our design and the rectangular design. (**b**) Simulated average fidelity as a function of MZI loss when the UMIs perform 20 × 20 unitary transformations. The fidelity values of the triangular and rectangular architectures are from [9].

In practice, both the tunable MZIs and the connection waveguides are lossy, which degrades the performance of the UMIs. There are two types of loss, namely, the balanced loss and the unbalanced loss. They depend on the path difference of all the paths in the interferometers. In our design, the propagation loss in an MZI is expected to mainly contribute to the balanced loss. For an *N* × *N* UMI, each path incorporates 3*N* − 1 MZIs to the maximum. Therefore, the balanced loss of our architecture increases as 3*N* − 1. The unbalanced loss in the interferometers degrades the fidelity of the transformations. As the insertion loss of the connection waveguide and the MZI is not necessarily the same, the path loss may be different for the interferometers. Therefore, we calculate the fidelity of the SCOW mesh-based UMI using the transfer matrix method. In each MZI, we add identical extra insertion loss to the two output ports. To compare the fidelity of our design with the triangular and rectangular designs, we use a similar procedure as mentioned in [9]. For a given *N*, we generate 500 groups of *ϕ_dq_* and *θ_dq_* with random values. With all the phase parameters of the MZIs, we can quantify the fidelity of the unitary transformation by our SCOW-based processor using the following equation [9],

(5) $$F\left(U_{\exp},U\right)={\left|\frac{tr\left(U^{†}U_{\exp}\right)}{\sqrt{N\times tr\left(U_{\exp}^{†}U_{\exp}\right)}}\right|}^{2}$$

where *U*_exp_ represents the transformation of a lossy SCOW processor and *U* represents the intended lossless unitary transformation. Figure 4a shows the average fidelity for a UMI changing with the size *N*, when the loss of the MZIs is set to 0.2 dB. We can see that the triangular architecture has the least tolerance to the unbalanced loss of the interferometers. As our design is originated from the rectangular architecture, the fidelity is only slightly lower than that of the rectangular design. The inset shows the close-up view of the fidelity of our design and the rectangular design. We can see that the fidelity values of our SCOW architecture are divided into two groups depending on the port numbers of the interferometers. The fidelity for odd number ports is lower than that for even number ports, because of the larger unbalanced loss in the interference paths. Figure 4b shows the fidelity as a function of MZI loss when the UMI size *N* is fixed to 20. As the insertion loss of one MZI is typically less than 0.5 dB, the fidelity of a 20 × 20 UMI based on the SCOW architecture is around 0.999 which is better than that of the triangular design. These simulation results show that our architecture has a high tolerance to the path loss difference in the interferometers. 

## 4. Conclusions

In summary, we proposed a two-dimensional SCOW mesh photonic processor to work as a rectangular UMI, which can accomplish arbitrary optical unitary transformation. Arbitrary unitary transformation has wide applications in quantum signal processing, photonic deep machine learning, etc. We presented the detailed adaptation synthesis algorithm for constructing an *N* × *N* rectangular UMI using the SCOW mesh-based photonic processor. The simulated fidelity shows that our architecture has a high tolerance to the path loss of the interferometers. This work extends the functionality of the SCOW mesh photonic processor, making it more powerful for multitude signal processing tasks.

## Figures and Tables

**Table 1:** Required number of MZIs to construct an *N* × *N* rectangular UMI.

UMI Scale *N*	SCOW Architecture
2*n* + 1	6*n*^2^ + 8*n* + 2
2*n*	6*n*^2^ + 5*n* − 1
